# Concordances and discrepancies between ICD-10 and DSM-IV criteria for anxiety disorders in childhood and adolescence

**DOI:** 10.1186/1753-2000-6-40

**Published:** 2012-12-26

**Authors:** Carmen Adornetto, Andrea Suppiger, Tina In-Albon, Murielle Neuschwander, Silvia Schneider

**Affiliations:** 1 Child- and Adolescent Psychiatry, University of Basel, Basel, Switzerland; 2 Department of Psychology, Clinical Psychology and Psychotherapy, University of Basel, Basel, Switzerland; 3 Universität Koblenz-Landau, Clinical Child and Adolescent Psychology, Koblenz-Landau, Germany; 4 Ruhr-Universität Bochum, Clinical Child and Adolescent Psychology, Universitätsstraße 150, Bochum, 44780, Germany

**Keywords:** ICD-10, DSM-IV-TR, Separation anxiety disorder, Specific phobia, Social phobia, Generalized anxiety disorder, Diagnostic criteria

## Abstract

**Background:**

Mental disorders are classified by two major nosological systems, the *ICD-10* and the *DSM-IV-TR*, consisting of different diagnostic criteria. The present study investigated the diagnostic concordance between the two systems for anxiety disorders in childhood and adolescence, in particular for separation anxiety disorder (SAD), specific phobia, social phobia, and generalized anxiety disorder (GAD).

**Methods:**

A structured clinical interview, the Kinder-DIPS, was administered to 210 children and 258 parents. The percentage of agreement, kappa, and Yule’s *Y* coefficients were calculated for all diagnoses. Specific criteria causing discrepancies between the two classification systems were identified.

**Results:**

*DSM-IV-TR* consistently classified more children than *ICD-10* with an anxiety disorder, with a higher concordance between *DSM-IV-TR* and the *ICD-10* child section (F9) than with the adult section (F4) of the *ICD-10*. This result was found for all four investigated anxiety disorders. The results revealed low to high levels of concordance and poor to good agreement between the classification systems, depending on the anxiety disorder.

**Conclusions:**

The two classification systems identify different children with an anxiety disorder. However, it remains an open question, whether the research results can be generalized to clinical practice since *DSM-IV-TR* is mainly used in research while *ICD-10* is widely established in clinical practice in Europe. Therefore, the population investigated by the *DSM* (research population) is not identical with the population examined using the *ICD* (clinical population).

## Background

Mental disorders are classified by two major nosological systems, *the International Classification of Diseases ICD*, [1] and the *Diagnostic and Statistical Manual of Mental Disorders DSM,*[2]. While the *DSM* is largely used for research purposes in Europe, European clinicians are mandated to report ICD codes [3]. Despite much effort to reduce the incompatibilities between the two classification systems [4], differences with respect to specific operationalization of many diagnoses still exist between the current versions, the *ICD-10*[1], last revision in 1993, and the *DSM-IV-TR*[2], last revision in 2000. A main conceptual difference between the two classification systems is how they differentiate anxiety disorders in adulthood and childhood. The *ICD-10* differentiates between anxiety disorders in childhood and in adulthood defining different criteria, whereas for the *DSM-IV-TR* this separation no longer exists. The subdivision of child-specific anxiety disorders is justified by the authors of the *ICD-10* because they argue that emotional disorders in infancy (1) discontinue until adulthood, (2) are rather reinforcements of normal developmental trends as independent, qualitatively unique phenomena, (3) differ from those of anxiety disorders in adulthood, and (4) cannot be clearly divided into more specific units, for instance phobic conditions [5].

Several studies investigated the agreement (percentage of cases with the same diagnosis on both classification systems) between various versions of the *ICD* and *DSM* as well as the concordance (percentage of cases with a positive diagnosis by both classification systems out of all cases with a positive diagnosis by either system) of mental disorders. The studies mainly focused on substance use e.g., [6-8], anxiety disorders, and affective disorders in adulthood e.g., [9-13]. The results of these studies show moderate to good agreements between the latest versions of *ICD* and *DSM*. However, considerable discrepancies nevertheless exist between the two systems.

Focusing on mental disorders in children and adolescents, two studies investigated the agreement between *ICD* and *DSM* diagnoses. Steinberger and Schuch [14] examined the diagnostic concordance for 66 children with obsessive-compulsive disorder (OCD) using semi-structured instruments, the International Diagnostic Checklists for *DSM-IV* and *ICD-10* IDCL, [15]. They found evidence of poor agreement between the two classification systems. While *DSM-IV* identified both children and adolescents with an OCD diagnosis, *ICD-10* particularly diagnosed adolescents. Since the *ICD-10* displayed an age-dependent concept of OCD, Steinberger and Schuch [14] argued that the *DSM-IV* criteria diagnosing OCD in childhood are superior to those of the *ICD-10*. Sorenson, Mors, and Thomsen [16] compared the two systems focusing on major depressive disorder, attention deficit hyperactivity disorder, and oppositional defiant disorder in a sample of 199 child psychiatric patients. The diagnoses were based on a semi-structured interview, the Schedule for Affective Disorders and Schizophrenia for Children-Present and Lifetime version [K-SADS-PL [17]. Sorenson et al. [16] found evidence of moderate agreement between the two systems. More children were diagnosed with a major depressive disorder using the *DSM-IV-TR* than when the *ICD-10* was applied. This discrepancy is mainly due to the fact that *ICD-10* requires depressed mood to be largely uninfluenced by circumstances. Also more children were diagnosed with ADHD according to the *DSM-IV*, because *ICD-10* does not offer a diagnosis covering ADHD subtypes such as hyperactive/impulsive or attention. The diagnosis for oppositional defiant disorder was interchangeable between the two systems.

In summary, there is evidence that the concepts of the *ICD* and *DSM* differ, resulting in discrepancies in the classification of children. More specifically, results of Sorenson et al. [16] and Steinberger et al. [14] provide preliminary evidence that the *DSM* diagnoses more children with a mental disorder than the *ICD-10*. However, to our knowledge, no study focusing on anxiety disorders, apart from OCD, in children and adolescents has been conducted so far. The aim of the present study was therefore to investigate the concordance of anxiety disorders in children and adolescents established by the *ICD-10* and the *DSM-IV-TR*. In addition, discrepancies at the diagnostic and the criterion level were determined for separation anxiety disorder (SAD), specific phobia, social phobia, and generalized anxiety disorder (GAD). Knowledge gained from this study may be essential for subsequent revisions of the *ICD-10* and *DSM-IV-TR* to reduce current differences between the two systems.

## Method

### Measures

Diagnoses were established with the Kinder-DIPS (**D**iagnostisches **I**nterview bei **P**sychischen **S**törungen des Kindes- und Jugendalters), a structured diagnostic interview that is designed to address both *ICD-10* and *DSM-IV* diagnostic criteria in children and adolescents [18]. The Kinder-DIPS consists of a child and a parent version (i.e., either the mother or father or both together) and assesses all anxiety disorders, depression, attention-deficit hyperactivity disorder, oppositional defiant disorder, sleep disorders, and eating disorders. The Kinder-DIPS shows a good reliability for anxiety disorders (child version: kappa = 0.88; parent version: kappa = 0.85) and other axis I disorders (child version, kappa = 0.48– 0.88, parent version, kappa = 0.85–0.94) [19].

### Participants and procedure

We collected data from 468 interviews (210 child and 258 parent interviews), which were conducted by seven psychologists (with a Master’s degree) and 28 Master’s students of clinical psychology. All interviewers received official standardized training in administering the Kinder-DIPS. Participants were recruited from the patient population at child and adolescent psychiatric clinics (*n* = 135) and from a research study at the University of Basel (*n* = 164). Insufficient knowledge of the German language was an exclusion criterion. The participating institutions recruited their patient samples. The patients at the University of Basel took part in a research program for anxiety disorders. Therefore, the recruitment at the University of Basel focussed on children with anxieties, especially on children with separation anxiety disorders. This explains the high prevalence rates of anxiety disorders as described in the result section. The interviewers administered the Kinder-DIPS to 210 children aged 6 to 17 years (*M* = 10.87, *SD* = 2.78; 51.4% boys) and to 258 parents of children aged 4 to 17 years (*M* = 9.77, *SD* = 3.08; 51.2% boys). The variance in the number of children and parents is a result of the fact that children can only be interviewed from the age of 6, whereas parent interviews can be conducted already when children are 4 years of age. The interview was part of the diagnostic assessment carried out at the participating institutions. Prior to the interview, children and parents were informed about the interview process and gave their written informed consent.

### Statistical analyses

The data were analysed with the Statistical Package for Social Sciences (SPSS) for Windows. We constructed 2x2 tables with the percentage of agreement for interrater reliability and the comparison of *ICD-10* (F4/F9) and *DSM-IV-TR* diagnoses for SAD (F93.0 vs. 309.21), specific phobia, phobic anxiety disorders of childhood, respectively (F40.2/F93.1 vs. 300.29), social phobia (F40.1/F93.2 vs. 300.23), and GAD (F41.1/F93.80 vs. 300.02). Kappa and Yule’s *Y* were calculated since kappa is not informative when base rates are low. Yule’s Y, on the other hand, is considered more robust [20]. Yule’s *Y* was calculated [21] for base rates lower than 10%. For cases that were negative on one diagnostic system and positive on the other, specific criteria causing the discrepancy were identified. The level of significance was set to 5%. We omitted the sections of the interview with missing values from further analyses. For the child interviews this resulted in a sample size reduction from 210 to 202 for the analyses of SAD, to 198 for specific phobia, to 186 for social phobia, and to 206 for GAD. For the parent interviews the sample size was reduced from 258 to 257 for the analyses of SAD, to 249 for specific phobia, to 228 for social phobia, and to 248 for GAD. The large sample size reduction for the analyses of social phobia is due to the interview process. We established a separate section for each diagnoses of social phobia (social phobia according to F4 in the *ICD-10*, social anxiety disorder of childhood according to F9 in the *ICD-10*, and *DSM-IV-TR* criteria*)*, since the criteria differ for these three diagnoses. The separation of the three sections ensures an exact assessment of the criteria. If an interviewer did not go through all three sections, because, for example, the criteria were not fulfilled in one section, the comparison could not be made.

## Results

### Interrater reliability

Differences between *ICD-10* and *DSM-IV-TR* diagnoses can be influenced by the reliability of the diagnoses. Therefore, we initially tested interrater reliability.

#### Child interviews

The testing included 136 interviews for SAD, 133 interviews for specific phobia, 122 interviews for social phobia, and 138 interviews for GAD. All examined anxiety disorders showed very good interrater reliabilities for *ICD-10* as well as for *DSM-IV-TR* diagnoses: kappa/Yule’s *Y* > 0.81 for SAD, Yule’s *Y* > 0.86 for specific phobia/phobic anxiety disorder, Yule’s *Y* = 1.00 for social phobia, and Yule’s *Y* = 1.00 for GAD. These findings provide a sufficient basis for the research question of this paper.

#### Parent interviews

We tested 169 interviews for SAD, 159 interviews for specific phobia, 142 interviews for social phobia, and 159 interviews for GAD. All examined anxiety disorders showed very good interrater reliabilities for *ICD-10* as well as for *DSM-IV-TR* diagnoses: kappa > 0.83 for SAD, Yule’s *Y* > 0.88 for specific phobia/phobic anxiety disorder, Yule’s *Y* > 0.85 for social phobia/social anxiety disorder, and Yule’s *Y* = 1.00 for GAD. Again, these findings justify the further study of the research question of this paper.

### Comparisons between the *ICD-10* and *DSM-IV-TR*

The following describes the *ICD-10* and *DSM-IV-TR* point prevalence rates as well as the level of concordance for each diagnosis. Further, we present criteria causing disagreements between the systems. A negative case (i.e., no diagnosis of disorders) may be identified, if several criteria are not fulfilled. The results of the comparisons are presented in Table 1. The table shows the cases diagnosed as having no anxiety disorder by both systems (DSM/ICD) (−/−) and those diagnosed with an anxiety disorder by either (−/+, +/−) or both (+/+) systems. Furthermore, the percentage of agreement between the two systems as well as the kappa and Yule’s *Y* coefficients of agreement are displayed.

**Table 1:** **Cross-classification of all anxiety disorders determined by the *DSM-IV-TR *and *ICD-10 *for child and parent interviews**

	**Frequencies**		**%**	**Kappa**	**Yule’s**	**Frequencies**		**%**	**Kappa**	**Yule’s**
	**−/−**	**−/+**			***Y***	**−/−**	**−/+**			***Y***
	**+/−**	**+/+**				**+/−**	**+/+**			
**Separation anxiety disorder (SAD)**										
*Child interviews*										
	ICD-10 F93.0									
DSM-IV-	164	2	92.6	.71	.85					
TR 309.21	13	23								
*Parent interviews*										
	ICD-10 F93.0									
DSM-IV-	160	4	89.9	.77	.84					
TR 309.21	22	71								
**Specific phobia/Phobic anxiety disorder of childhood**										
*Child interviews*										
	ICD-10 F40.2					ICD-10 F93.1				
DSM-IV-	167	7	87.8	(.31)	.52	170	4	88.9	(.30)	.58
TR 300.29	17	7				18	6			
*Parent interviews*										
	ICD-10 F40.2					ICD-10 F93.1				
DSM-IV-	201	7	84.3	.25	.43	207	1	88.7	.45	.82
TR 300.29	32	9				27	14			
**Social phobia/Social anxiety disorder of childhood**										
*Child interviews*										
	ICD-10 F40.1					ICD-10 F93.2				
DSM-IV-	175	-	94.6	(.16)	1.00	175	-	94.1	(−)	-
TR 300.23	10	1				11	-			
*Parent interviews*										
	ICD-10 F40.1					ICD-10 F93.2				
DSM-IV-	196	1	87.3	(.15)	.64	192	5	89.0	.41	.64
TR 300.23	28	3				20	11			
**Generalized anxiety disorder (GAD)**										
*Child interviews*										
	ICD-10 F41.1					ICD-10 F93.80				
DSM-IV-	195	1	95.7	(.30)	.75	196	-	96.1	(.32)	1.00
TR 300.02	8	2				8	2			
*Parent interviews*										
	ICD-10 F41.1					ICD-10 F93.80				
DSM-IV-	229	5	93.9	(.32)	.62	234	-	98.8	(.87)	1.00
TR 300.02	10	4				3	11			

*Note.* Kappa coefficients, which depict an underestimation due to the low base rate of less than 10% are reported in parentheses. (−/−) shows the cases diagnosed as having no anxiety disorder by both systems (DSM-IV/ICD-10), (−/+, +/−) shows those diagnosed with an anxiety disorder by either or both (+/+) systems. DSM-IV diagnosis is presented first, then the ICD-10 diagnosis.

### Separation anxiety disorder (SAD) ICD-10 F93.0 and DSM-IV-TR 300.21

#### Child interviews

We found 12.4% prevalence for the *ICD-10* and 17.8% for the *DSM-IV-TR* diagnosis. Thirty-eight children met the criteria for SAD on either classification system with a concordance of 63.9%. Two cases were positive on *ICD-10* and negative on *DSM-IV-TR*. In one case this was due to the fact that the child did not exhibit three or more characteristics of *DSM* criterion and in the other case because the child did not show clinically significant impairment or distress (*DSM* criterion D). 16 children received a positive diagnosis with the *DSM-IV-TR* while the diagnosis based on the *ICD-10* was negative: of these, 3 children lacked three or more characteristics of *ICD* criterion A, and in 13 children the onset of the anxiety was after the age of 6 (*ICD* criterion C).

#### Parent interviews

The prevalence for the *ICD-10* was 29.2% and 36.2% for the *DSM-IV-TR*. Ninety-seven children were diagnosed with SAD on either classification system with a concordance of 73.2%. Four children were positive on *ICD-10* and negative on *DSM-IV-TR* because they did not show clinically significant impairment or distress (*DSM* criterion D). 24 children received a positive diagnosis with the *DSM-IV-TR* diagnosis while the *ICD-10* diagnosis was negative. Two of the 24 children lacked three or more characteristics of *ICD* criterion A while 6 children were diagnosed with a GAD (*ICD* criterion B). By 16 children the anxiety became evident after the age of 6 (*ICD* criterion C).

### Specific phobia ICD-10 F40.2 and DSM-IV-TR 300.29

#### Child interviews

The *ICD-10* prevalence was 7.1%, the *DSM-IV-TR* prevalence being 12.1%. Thirty-one children met the criteria for specific phobia on either classification system with a concordance of 22.6%. Eight children received a positive *ICD-10* diagnosis while their *DSM-IV-TR* diagnosis was negative: of these, 2 children almost always never showed a fearful reaction in the phobic situation (*DSM* criterion B), 5 children did not demonstrate clinically significant impairment or distress (*DSM* criterion E), and one of the children had experienced fear for less than 6 months (*DSM* criterion F). For 24 children the *DSM-IV-TR* diagnosis was positive and the *ICD-10* was negative; 11 of the children did not fulfil the essential panic symptoms (*ICD* criterion B), and 13 children did not have significant emotional distress and insight (*ICD* criterion C).

#### Parent interviews

Our analysis revealed a prevalence of 6.4% for the *ICD-10* and 16.5% for the *DSM-IV-TR*. Forty-eight children met the criteria for specific phobia on either classification system with a concordance of 18.7%. Seven of the children received a positive diagnosis according to the *ICD-10* while their diagnosis based on the *DSM-IV-TR* negative. Two of the seven children almost always never experienced a fearful reaction in the phobic situation (*DSM* criterion B) and 5 children had no clinically significant impairment or distress (*DSM* criterion E). The *DSM-IV-TR* diagnosis was positive for 43 children while they received a negative diagnosis based on the *ICD-10*. 19 children of these 43 lacked the essential panic symptoms (*ICD* criterion B) and 24 children reported that they did not experience significant emotional distress and insight (*ICD* criterion C).

### Specific phobia ICD-10 F93.1 (Phobic anxiety disorder of childhood) and DSM-IV-TR 300.29

#### Child interviews

The results showed a prevalence of 5.1% for the *ICD-10* and 12.1% for the *DSM-IV-TR*. Twenty-eight children met the criteria for specific phobia on either classification system with a concordance of 21.4%. Four children received a positive *ICD-10* diagnosis while based on the *DSM-IV-TR their diagnosis was* negative. Three of these 4 children were positive on *ICD-10* and negative on *DSM-IV-TR* since they almost always never showed a fearful reaction in the phobic situation (*DSM* criterion B). For one child the duration of the fear was less than 6 months (*DSM* criterion F). Eighteen children were given a positive diagnosis according to the *DSM-IV-TR* while their *ICD-10* diagnosis was negative. Seventeen of the 18 children were not socially impaired (*ICD* criterion A) and 1 child was diagnosed with GAD (*ICD* criterion B).

#### Parent interviews

We found an *ICD-10* prevalence of 6.0% and a *DSM-IV-TR* prevalence of 16.5%. Forty-two children met the criteria for specific phobia on either classification system with a concordance of 33.3%. Only one case was positive on *ICD-10* and negative on *DSM-IV-TR* because the child almost never showed a fearful reaction in the phobic situation (*DSM* criterion B). Twenty-eight children were granted a positive *DSM-IV-TR* diagnosis, the *ICD-10* diagnosis being negative. Twenty-six of the 28 children did not fulfil the criteria for being socially impaired (*ICD* criterion A) while 2 children were diagnosed with GAD (*ICD* criterion B).

### Social phobia ICD-10 F40.1 and DSM-IV-TR 300.23

#### Child interviews

Our results demonstrated an *ICD-10* prevalence of 0.5% while prevalence of the *DSM-IV-TR* was 5.9%. Eleven children met the criteria for social phobia on either classification system with a concordance of 9.1%. None of the children was positive on the *ICD-10* and negative on the *DSM-IV-TR*. Thirteen children received a positive *DSM-IV-TR* diagnosis while being negative based on the *ICD-10*. Ten of the children lacked the necessary panic symptoms (*ICD* criterion B) while 3 children did not have significant emotional distress and insight (*ICD* criterion C).

#### Parent interviews

The prevalence for the *ICD-10* was 1.8% and 13.6% for the *DSM-IV-TR*. Thirty-two children met the criteria for social phobia on either classification system with a concordance of 9.4%. One child was positive on *ICD-10* and negative on *DSM-IV-TR* because of the lack of clinically significant impairment or distress (*DSM* criterion E). Positive *DSM-IV-TR* diagnosis and negative *ICD-10* diagnosis was given to 45 children. Twenty-five of them did not demonstrate the necessary panic symptoms (*ICD* criterion B) and 20 children lacked significant emotional distress and insight (*ICD* criterion C).

### Social phobia ICD-10 F93.2 (Social anxiety disorder of childhood) and DSM-IV-TR 300.23

#### Child interviews

The prevalence for the *DSM-IV-TR* was 5.9%. Eleven children met the criteria for social phobia on *DSM-IV-TR*. None of the children received a positive diagnosis on *ICD-10*. Twenty-seven children received a positive *DSM-IV-TR* and a negative *ICD-10* diagnosis. Eight children out of the 27 did not meet *ICD* criterion A, which requires social fear and avoidance, 2 children were not embarrassed or worried about their behaviour toward strangers (*ICD* criterion B) and 11 children did not have clinically significant impairment or distress (*ICD* criterion C). One child did not have satisfying relationships with family members and friends (*ICD* criterion D), and for 5 children the onset of the anxiety was after the age of 6 (*ICD* criterion E).

#### Parent interviews

*ICD-10* had a prevalence of 7.0% for and the *DSM-IV-TR* of 13.6%. Thirty-six children met the criteria for social phobia on either classification system with a concordance of 30.6%. All five children who were positive on *ICD-10* and negative on *DSM-IV-TR* did not display fear and humiliation (*DSM* criterion A). Thirty-six children received a positive *DSM-IV-TR* diagnosis and were diagnosed negative according to *ICD-10*. From the 36 children, 12 children did not display social fear and avoidance (*ICD* criterion A), 2 children were not embarrassed or worried about their behaviour toward strangers (*ICD* criterion B), 16 children did not have clinically significant impairment or distress (*ICD* criterion C), 2 children did not have satisfying relationships with family members and friends (*ICD* criterion D), and for 3 children the onset of the anxiety was after the age of 6 (*ICD* criterion E), while 1 child was diagnosed with GAD (*ICD* criterion F).

### Generalized anxiety disorder (GAD) ICD-10 F41.1 and DSM-IV-TR 300.02

#### Child interviews

The prevalence for the *ICD-10* was 1.5% and for the *DSM-IV-TR* 4.9%. Eleven children met the criteria for GAD on either classification system with a concordance of 18.2%. One child was positive on *ICD-10* and negative on *DSM-IV-TR* because the child did not exhibit the required symptoms (*DSM* criterion C). All 8 children, who were positive on *DSM-IV-TR* and negative on *ICD-10,* did not display the required symptoms (*ICD* criterion B).

#### Parent interviews

The prevalences for the *ICD-10* and for the *DSM-IV-TR* were 3.6% and 5.6% respectively. Nineteen children met the criteria for GAD on either classification system with a concordance of 21.1%. Nine children were diagnosed positive on the *ICD-10* and negative on the *DSM-IV-TR*. Two out of the 9 children had no difficulty controlling worry (*DSM* criterion B), 4 children lacked the required symptoms (*DSM* criterion C), and 3 children did not have clinically significant impairment or distress (*DSM* criterion E). All 10 children, who were positive on *DSM-IV-TR* and negative on *ICD-10,* did not demonstrate the required symptoms (*ICD* criterion B).

### Generalized anxiety disorder (GAD) ICD-10 F93.80 and DSM-IV-TR 300.02

#### Child interviews

We found a prevalence of 1.0% for the *ICD-10* and 4.9% for the *DSM-IV-TR*. Ten children met the criteria for GAD on either classification system with a concordance of 20%. Ten children received a positive diagnosis based on the *DSM-IV-TR* and were diagnosed negative on the *ICD-10*. Eight out of these 10 children did not display the required symptoms (*ICD* criterion C) and 2 children did not show worry in at least two situations (*ICD* criterion D).

#### Parent interviews

The *ICD-10* prevalence was 4.4% for and the prevalence for *DSM-IV-TR* was 5.6%. Fourteen children met the criteria for GAD on either classification system with a concordance of 78.6%. Three children did not exhibit the required physical symptoms (*ICD* criterion C), while their *DSM-IV-TR* diagnosis was positive and *ICD-10* diagnosis was negative.

## Discussion

The present study describes an investigation of concordance between *ICD-10* and *DSM-IV-TR* diagnoses of anxiety disorders in children and adolescents, specifically for separation anxiety disorder (SAD), specific phobia, social phobia, and generalized anxiety disorder (GAD). The results indicated low to high levels of concordance and poor to good agreement between the classification systems, depending on the anxiety disorder. As seen by the high interrater reliability of the established diagnoses in the present study, the disagreement between the two systems is unlikely to be the result of unreliable diagnostic processes.

Regarding the child interviews, the agreement between diagnoses established with the *ICD-10* and the *DSM-IV-TR* was good for SAD (kappa = 0.71), unsatisfactory for specific phobia (F40.2: Yule’s *Y* = 0.52) and for phobic anxiety disorder of childhood (F93.1: Yule’s *Y* = 0.58), and satisfactory for GAD (F41.1: Yule’s *Y* = 0.75). For social phobia (F40.1), social anxiety disorder of childhood (F93.2), and GAD (F93.80) no cases were diagnosed as positive only by the *ICD-10*.

The results for the parent interviews show very good agreement for SAD (kappa = 0.77), and satisfactory agreement for phobic anxiety disorder of childhood (F93.1: kappa = 0.45) and social anxiety disorder of childhood (F93.2: kappa = 0.41). The agreement was poor for specific phobia (F40.2: kappa = 0.25), and unsatisfactory for social phobia (F40.1: Yule’s *Y* = 0.64) and GAD (F41.1: Yule’s *Y* = 0.62). For GAD (F93.80), no cases were diagnosed as positive only by the *ICD-10*.

Good agreement between the *ICD-10* and *DSM-IV-TR* was found for all negative diagnoses. Focussing on the positive diagnoses, the *DSM-IV-TR* consistently classified more children with an anxiety disorder than the *ICD-10* for SAD, GAD, social and specific phobia. Results are comparable to previous studies comparing *ICD-10* and *DSM-IV* diagnoses of mental disorders in children and adolescents [14,16].

The concordance was higher for the *DSM-IV-TR* and the *ICD-10* child section (F9) than for the *ICD-10* adult section (F4), especially for the parent interviews. The highest level of concordance was found for SAD (child interviews: 63.9%; parent interviews: 73.2%) and GAD (F93.80; parent interviews: 78.6%). However, there was only a low level of concordance for GAD (F93.80) in the child interviews (20%) and also for GAD (F41.1) in the child and parent interviews (18.2%, 21.1%). This result is due to the stricter criterion C in the *ICD-10* than in the *DSM-IV-TR* requiring three symptoms to be associated with anxiety and worry (e.g., muscle tension, sleep disturbances, restlessness, irritability). However, in DSM-IV-TR only one physical symptom for a diagnosis of GAD in children is required. The revision of the DSM-V ( http://www.dsm5.org) points out that there is limited evidence for the threshold of three or more symptoms associated with anxiety and worry. Therefore, it is proposed that, even for adults, one or more associated symptoms should be sufficient for a diagnosis of GAD. A low level of concordance was found for specific phobia (F40.2), phobic anxiety disorder of childhood (F93.1), social phobia (F40.1), and social anxiety disorder of childhood (F93.2) in the child and parent interviews.

### Discrepancies on criterion level between the *ICD-10* (F4) and the *DSM-IV-TR*

Regarding discrepancies on criterion level between the *ICD-10* (F4) and the *DSM-IV-TR,* the low concordance for specific and social phobias was particularly due to the *ICD-10* criterion requiring significant emotional distress and insight. Thereby, the demand for insight was mainly not fulfilled. When *ICD-10* positive cases were negative on the *DSM-IV-TR,* the main reason was that the *DSM-IV-TR* criterion requires clinically significant impairment or distress. Some children and parents denied impairment or distress due to the fear or avoidance as defined in the *DSM-IV-TR,* however, at the same time they indicated significant emotional distress concerning the panic symptoms described in the *ICD-10*.

### Discrepancies on criterion level between the *ICD-10* (F9) and the *DSM-IV-TR*

Discrepancies in SAD diagnoses were mainly due to *ICD-10* criterion B requiring the exclusion of GAD and criterion C requiring onset before the age of 6. For SAD, *DSM-IV-TR* requires an onset before age 18 years. *DSM-V* is considering deleting the specifier “early onset before age 6 years” as there is no evidence to justify such a specifier ( http://www.dsm5.org). Furthermore, the retrospective NCS-R study showed a median age of onset for SAD at 7 years of age [22] supporting a less strict criterion for SAD in *ICD-10*.

When the *DSM-IV-TR* diagnosis was negative, this was mainly due to the criterion D requiring clinically significant impairment or distress. With regard to specific and social phobias, discrepancies were in particular due to the criteria defining the fear and impairment. For specific phobia in the *ICD-10*, criterion A requires fear with social impairment, whereas the *DSM-IV-TR* criterion A requires only fear. The type of impairment is more widely defined in the *DSM-IV-TR* (criterion E; e.g. impairment in social, occupational, or other important areas of functioning). For social phobia, in the *ICD-10* social fear and avoidance are required (criterion A), whereas in the *DSM-IV-TR* social fear and feelings of humiliation are required (criterion A). Further, the feature of impairment is much more clearly defined in the *ICD-10* (criterion C) than in the *DSM-IV-TR* (criterion E). The main reason for a negative *DSM-IV-TR* diagnosis was criterion A (marked and persistent fear of one or more social or performance situations).

In sum, the low level of concordance is particularly due to the two systems using different definitions of common features. More specifically, the definitions of (specific and social) fear and impairment differ significantly.

Kendell [23] recommends that minor points of difference between the two classification systems should be revised to become identical, and if the differences are substantial, the validity of each system should be assessed. The results of the present study indicate that concordance between the child section (F9) in the *ICD-10* and the *DSM-IV-TR* might be improved by a less strict formulation of the *ICD-10* criteria. More specifically, the onset of SAD, the definition of impairment and insight for specific and social phobia, and the required number of essential symptoms associated with anxiety and worry for GAD should be changed in *ICD-11*. In addition, the conceptualization of GAD (F93.80) in the *ICD-10* is an important issue to consider. According to the *ICD-10,* GAD is conceptualized higher in the hierarchy of anxiety disorders in childhood, since a diagnosis of GAD is an exclusion criterion for other anxiety diagnoses in childhood (F9). However, there are no empirical studies to show that diagnoses of SAD, specific, and social phobia could not be comorbid with GAD. Therefore in *ICD-11*, it should be possible to assign comorbid anxiety disorders with GAD. However, it has to be acknowledged that the diagnosis of GAD is considered difficult and not reliable, due to high rates of coexisting disorders and the overlap in symptomatology, e.g., depression and other anxiety disorders [24], therefore diagnostic criteria will most likely be changed again for *DSM-V*. How comorbidity is dealt with by the DSM-IV-TR and the ICD-10 is actually quite similar, since both systems with their descriptive approaches allow multiple diagnoses [2].

The results of the present study support the proposed change in *DSM-V* to delete the age criteria for SAD. Regarding the discussion to separate functional impairment and diagnoses [25], the good agreement for all negative diagnoses in this study underlines the importance of integrating the criteria of overall impairment and distress in assigning diagnoses.

It is important to note that the *ICD* is used in clinical practice in Europe, whereas the *DSM* is the most frequently used system for research purposes [26]. The differences between the two systems lead to different diagnoses in clinical practice and research. Knowledge gained from research may therefore not be directly applicable to clinical practice. If the population investigated by the *DSM* (research population) is not identical with the population examined by the *ICD* (clinical population), it is open to question whether the results from research can be directly applicable to clinical practice. This is an important issue considering the fact that psychotherapy and psychopharmacology treatment research is usually based on *DSM* diagnoses. The *ICD-10* differentiation between anxiety disorders in adulthood and childhood leads to diagnostic problems in clinical practice; for instance it is unclear which diagnoses a 17-year old adolescent, who fulfils criteria for both specific phobias (F4 and F9), should receive. In addition, the argument of discontinuing disorders of *ICD-10* is not empirically supported. On the contrary, several studies indicate that anxiety disorders in childhood are a major risk factor for the development of further mental disorders [27-29]. Suggesting that *ICD-10* classifies fewer children with anxiety disorders than *DSM-IV-TR,* indicates that these children are not recognized, and therefore they remain untreated and are at risk to develop further mental disorders.

Some shortcomings of the present study have to be acknowledged. The small sample size, with rare positive diagnoses, especially for social phobia and GAD, should be mentioned as a limitation. The sample size may have been too small to detect rarely occurring discrepancies. To confirm the reported results, studies with a larger sample size including more positive diagnoses are necessary. The focus of the present study was on concordance and discrepancies on the criterion level and no conclusions about the validity of the diagnoses were drawn. It would be useful for future research to examine diagnostic validity, in particular for diagnostic categories with low concordance.

In sum, the low level of concordance is due to substantial differences in criteria for anxiety disorders between the classification systems. The two systems contain different concepts, and therefore classify different children. This is problematic, as children with significant problems and impairments may remain undiagnosed and therefore untreated. Consequently, the goal for both DSM-V and ICD-11 should be to diagnose children as adequately as possible, get them into treatment and so reduce further distress. Therefore, each diagnostic criterion should be empirically investigated. In addition, comparable diagnostic criteria would lead to consistent prevalence rates regardless of the classification system used.

## Competing interest

The authors declare that they have no competing interests.

## Authors’ contributions

CA carried out interviews, the analyses, and drafted the manuscript. AS has made substantial contribution to acquisition of data. TI contributed to acquisition of data, the drafting and revising of the manuscript. MN participated in the interpretation of the data and revising the manuscript. SS made substantial contributions to the design of the study, the interpretation of the data, drafting and revising the manuscript. All authors read and approved the final manuscript.
